# Combined Immunodeficiency in Patients With Trichohepatoenteric Syndrome

**DOI:** 10.3389/fimmu.2018.01036

**Published:** 2018-05-11

**Authors:** Frédéric Vély, Vincent Barlogis, Evelyne Marinier, Marie-Edith Coste, Béatrice Dubern, Emmanuelle Dugelay, Julie Lemale, Christine Martinez-Vinson, Noël Peretti, Ariane Perry, Patrice Bourgeois, Catherine Badens, Olivier Goulet, Jean-Pierre Hugot, Catherine Farnarier, Alexandre Fabre

**Affiliations:** ^1^Aix Marseille Univ, CNRS, INSERM, CIML, Marseille, France; ^2^APHM, Hôpital de la Timone, Service d’Immunologie, Marseille-Immunopôle, Marseille, France; ^3^APHM, Hôpital de la Timone, Service d’Hématologie et Oncologie Pédiatrique, Marseille, France; ^4^APHP Robert Debré, Department of Pediatric Gastroenterology, Hepatology and Nutrition, Paris, France; ^5^APHM, Hôpital de la Timone Enfant, Service de Pédiatrie Multidisciplinaire, Marseille, France; ^6^Nutrition et Gastroentérologie Pédiatriques, Hôpital Armand-Trousseau, UMR-S U1166 Nutriomics, UPMC, Sorbonne University, Paris, France; ^7^Department of Pediatric Nutrition, University Pediatric Hospital of Lyon, Hospices Civils de Lyon HCL, INSERM U1060, CarMeN Laboratory, University Claude Bernard Univ Lyon-1, Lyon, France; ^8^APHP, Hôpitaux Universitaires Paris Sud, Hôpital Antoine Béclère, Centre de référence des maladies héréditaires du métabolisme hépatique, Clamart, France; ^9^APHM, Hôpital de la Timone Enfant, Service de biologie moléculaire, Marseille, France; ^10^Aix Marseille Univ, INSERM, MMG, Marseille, France; ^11^APHP, Necker-Enfants Malades Hospital, Department of Pediatric Gastroenterology, Hepatology and Nutrition, Paris-Descartes University, Intestinal Failure Rehabilitation Center, National Reference Centre for Rare Digestive Diseases, Paris, France; ^12^APHP, Hôpital Robert Debré, Paris, France; ^13^UMR 1149, Institut National de la Santé et de la Recherche Médicale, Paris, France; ^14^Labex Inflamex, Université Paris-Diderot Sorbonne Paris-Cité, Paris, France

**Keywords:** SKIV2L, TTC37, syndromic diarrhea/trichohepatoenteric syndrome, immunodeficiency, memory B cells, NK cells, T cells

## Abstract

The syndromic diarrhea/trichohepatoenteric syndrome (SD/THE) is a rare and multi-system genetic disorder caused by mutation in *SKIV2L* or in *TTC37*, two genes encoding subunits of the putative human SKI complex involved in RNA degradation. The main features are intractable diarrhea of infancy, hair abnormalities, facial dysmorphism, and intrauterine growth restriction. Immunologically this syndrome is associated with a hypogammaglobulinemia leading to an immunoglobulin supplementation. Our immune evaluation of a large French cohort of SD/THE patient revealed several immunological defects. First, switched memory B lymphocytes count is very low. Second, IFN-γ production by T and NK cells is impaired and associated with a reduced degranulation of NK cells. Third, T cell proliferation was abnormal in 3/6 *TTC37*-mutated patients. These three patients present with severe EBV infection and a transient hemophagocytosis which may be related to these immunological defects. Moreover, an immunological screening of patients with clinical features of SD/THE could facilitate both diagnosis and therapeutic management of these patients.

## Introduction

Syndromic diarrhea/trichohepatoenteric syndrome (SD/THE) was initially described by Girault et al. in 1994 (1). It is a severe and rare disorder that is characterized by intractable diarrhea, facial and hair abnormalities, intrauterine growth retardation, skin abnormalities, liver disease, and less frequently congenital cardiac defects and platelet anomaly (2). A molecular basis of SD/THE has been reported recently. THE syndrome 1 (OMIM, #222470; *614589) and THE syndrome 2 (#614602; *600478) are caused by mutations in the genes encoding the tetratricopeptide repeat domain-containing protein 37 (*TTC37*) and superkiller viralicidic activity 2 (*SKIV2L*) genes, respectively. TTC37 and SKIV2L are both components of the superkiller (SKI) complex (3). The superkiller complex is a cofactor of the cytosolic exosome, which is involved in the degradation of aberrant mRNA molecules (4). The mechanism by which a defect in the mRNA degradation system leads to the symptoms associated with SD/THE remains unclear. Since its initial description, immunological defects such as low serum concentration of immunoglobulins and decreased or absent antibody responses to vaccination have been reported (2, 5, 6).

The clinical evolution of a large French cohort of SD/THE patients has been previously described (7). Although most of these patients were administered antibody replacement therapy, 20% still died from infection, one from measles. For this reason, we investigated more deeply the B, T, and NK cell compartments to better understand the immunological defects of THES1 and THES2 patients.

## Results

### Patients Presentation

We collected immunological data of nine patients with SD/THE syndrome caused by mutations of either *TTC37* or *SKIV2L* which is a large cohort regarding this rare deficiency. This immunological study was performed in the Immunology Department of the Timone Hospital during patients’ follow-up in four French Hospitals. All patients presented with a classical SD/THE phenotype: intractable diarrhea of infancy beginning at a median age of 31 day (0–335), need for a parenteral nutrition, hair abnormalities, and trichorhexis nodosa (8/9). Intrauterine growth restriction or small size for gestational age was noted for 7/9 patients with a median birth weight of 1,580 g (1,000–2,950). Skin abnormalities were noted for 7/8 patients and facial dysmorphy for 6/8 patients. Finally, about half of the patients had hepatic abnormalities (5/8). Three patients harbored homozygous or compound heterozygous mutation in *SKIV2L* versus six patients with homozygous or compound heterozygous mutation in *TTC37*. All these variants were previously reported (2, 3). These patients presented a good nutritional status at the time of the sample, but only patients four and five were weaned of parenteral nutrition. The details of the clinical presentation of the patients are shown in the Table 1.

**Table 1:** Patients characteristics.

	Patient 1	Patient 2	Patient 3	Patient 4	Patient 5	Patient 6	Patient 7	Patient 8	Patient 9
Mutated gene	SKIV2L	SKIV2L	SKIV2L	TTC37	TTC37	TTC37	TTC37	TTC37	TTC37
Mutation 1	c.848 G>A p.Trp283*	c.3561_3581del p.Ser1189_Leu1195del	c.1635_1636insA p.Gly546Arg fs*35	c.287_291del p.Leu96Trp fs*11	c.287_291del p.Leu96Trp fs*11	c.1708 C>T p.Arg570*	c.2515+1 G>C p.Cys813Val fs*56	c.3015−1 G>A	c.3808 C>G p.Pro1270Ala
Mutation 2	c.1022 T>G p.Val341Gly	c.3561_3581del p.Ser1189_Leu1195del	c.1635_1636insA p.Gly546Arg fs*35	c.287_291del p.Leu96Trp fs*11	c.287_291del p.Leu96Trp fs*11	c.3185_3201dup p.Lys1068Ser fs*2	c.2515+1 G>C p.Cys813Val fs*56	c.4454 T>G p.Leu1485Arg	c.3808 C>G p.Pro1270Ala
Intractable diarrhea	Y	Y	Y	Y	Y	Y	Y	Y	Y
Onset of diarrhea	D31	D21	0	D21	D335	D72	D204	D31	D19
Small for gestationnal age	Y	Y	Y	N	N	Y	Y	Y	Y
Hair abnormalities^a^	Y	Y	Y	Y	Y	Y	Y	Y	Y
Facial dysmorphy^a^	N	U	Y	Y	N	Y	Y	Y	Y
Liver disease	Y	U	N	N	N	Y	Y	Y	Y
Dermatological abnormalities	Y	U	Y	Y	N	Y	Y	Y	Y
Cardiac abnormalities	N	N	N	N	N	N	N	N	N
Hemophagocytosis	N	N	N	Y (EBV)	Y (EBV)	Y (Kawasaki disease)	Y (EBV)	N	N
Immunoglobulin replacement therapy (period)	N	Y (D99–328)	Y (D33–4,023)	N	Y (D336–1,858)	N	Y (1,009–on going)	Y (D202–on going)	Y (D97–on going)
Ig classes and subclasses and age at the diagnosis	D253	D99	D181	D45		D153	D395	D153	D60
IgA level (g/l)	0.56	0.19	0.07	<0.27		1.80	0.32	0.13	<0.04
IgG level (g/l)	4.10	1.31	1.85	3.46		10.25	4.90	1.36	0.44
IgM level (g/l)	0.50	0.20	0.30	0.61		0.50	0.57	1.5	0.05
IgG1 level (g/l)	2.99	<0.79					4.68	3.1	
IgG2 level (g/l)	0.48	<0.19					0.59	0.86	
IgG2 level (g/l)	0.87	0.126					0.82	1.16	
IgG4 level (g/l)	0.05	0.02					<0.01	0.04	
Virus infection			EBV primoinfection requiring Mabthera, persistent EBV replication	EBV	EBV	Adenovirus infection requiring hospital admission in the PICU	EBV		

*Y, yes; N, no; U, unknown; PICU, pediatric intensive care unit*.

*^a^Facial and hair abnormalities are nearly constant feature of THE syndrome. Most of the time, hair is described as woolly, uncombable, easily removable, and poorly pigmented, and the trichohexis nodosa is found when it is sought. Facial dysmorphia is less noticeable, and it may become more evident over time. The main feature is coarse facial expressions with huge forehead and broad nasal root*.

A written informed consent was obtained from each participant for the publication. This study was performed according to French ethics policy (Art. L. 1243-1 et Art. L. 1245-2 du Code de la Santé Publique).

### Drastic Reduction of the Circulating Switched Memory B Cells

**Figure 1:**
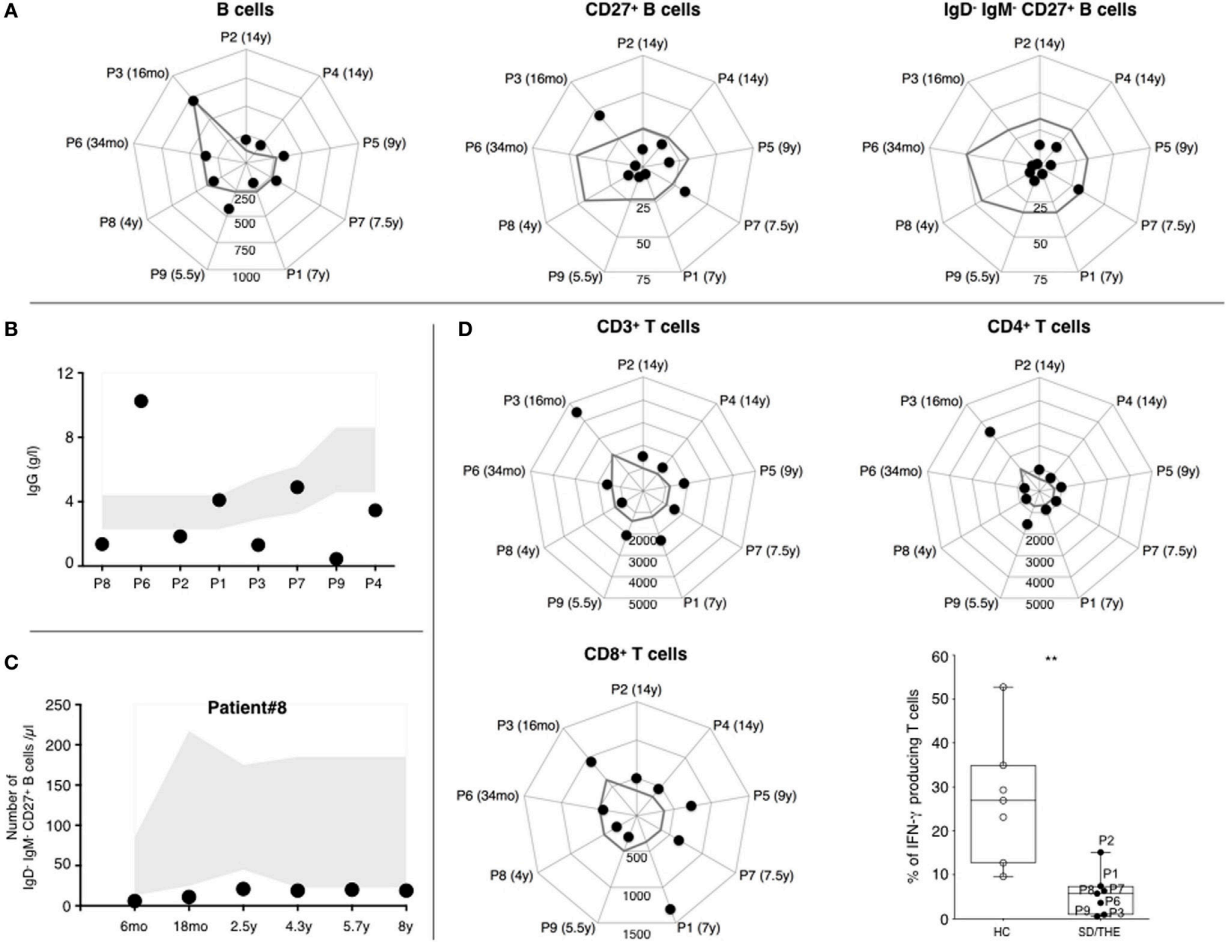
Phenotypic and functional analysis of B and T lymphocytes. **(A)** Absolute numbers of CD19^+^ B lymphocytes, CD19^+^CD27^+^ memory B lymphocytes, and CD19^+^IgM^−^IgD^−^CD27^+^ switched memory B lymphocytes (cells per μl). **(B)** IgG level at the age of diagnosis. Normal values are indicated as a gray area. **(C)** 8-years follow-up of B cell subsets in a syndromic diarrhea/trichohepatoenteric syndrome (SD/THE) patient. Absolute numbers of CD19^+^IgM^−^IgD^−^CD27^+^ switched memory B lymphocytes (cells per μl). Normal values are indicated as a gray area. **(D)** Counts of total CD3^+^, CD3^+^CD4^+^, and CD3^+^CD8^+^ T cell subsets (cells per μl). The gray line indicates the lower limit of normal values according to the age. T cell functions evaluated by their ability to produce interferon gamma (lower right panel) after PMA/ionomycin activation as previously described (9). Healthy controls (*n* = 7) and patients (*n* = 7) are indicated as open circles and closed circles, respectively. Box-plot with medians, 25th–75th percentiles, and min–max values are shown. ns, not significant, *p* > 0.05; *p < 0.05; **p < 0.01; ***p < 0.001; ***p < 0.001; ***p < 0.001 (Mann–Whitney test).

First, 3/6 THES1 patients and 2/3 THES2 patients displayed serum IgG values below age-appropriate normal ranges (Figure 1B). Our results showed that this humoral defect was not systematically associated with a B cell lymphopenia. CD19^+^ B cell counts were mostly at the lower limit of the normal ranges (Figure 1A, left panel) (8). Only 2/6 THES1 patients and 1/3 THES2 patient present with low B cell count at the time of diagnosis. A reduced number of CD27^+^ memory B cells was found in all but two patients (Figure 1A, middle panel). Moreover, it appears that the number of switched memory IgM^−^CD27^+^ B cells was deeply reduced in all patients except for one THES1 patient who had a slight decline (Figure 1A, right panel). Interestingly, this B cell subset was reduced for the last 8 years in one patient for whom a longitudinal monitoring was performed (Figure 1C).

### Specific Antibody Production Is Highly Variable After Vaccination

The evaluation of specific responses after vaccination was made difficult by the implementation of an early immunoglobulin replacement therapy. This information was not available for patients 1, 2, 5, 8, and 9. For patient 3, anti-diphtheria and anti-poliovirus responses were negative, whereas anti-tetanus antibodies were found protective 62 months after the last of seven immunization shots. Detections of anti-tetanus and anti-diphtheria antibodies were positive, whereas anti-polio antibodies were negative in patient 4 at the age of 18 months. Three years after the last of four shots in patient 6, anti-diphtheria and anti-tetanus antibodies were negative, whereas anti-measles, anti-mumps, and anti-rubella antibodies were positive. For patient 7, anti-diphtheria response was positive 1 month after the fifth injection. By contrast, B cells in this patient failed to produce specific antibodies directed against tetanus, polio, haemophilus, measles, rubella, and mumps antigens after vaccination.

### T Cells Can Proliferate but Produce a Small Amount of IFN-γ

As for B cells, T cell counts in patients were mostly at the lower limit of the normal ranges (Figure 1D, upper left panel) (8). There was no major difference in CD4^+^ and CD8^+^ T cell distribution (Figure 1D). The distribution between CD45RO^+^ memory and CD45RA^+^ naive CD4^+^ T cells was preserved (data not shown). T-cell proliferation was monitored by quantifying CFSE-dilution within peripheral blood mononuclear cell (PBMC) after activation with either mitogens such as phytohemagglutinin (PHA), anti-CD3 antibodies, or antigens (tetanus toxoid, tuberculin, and candidin). Both antigen- and mitogen-induced proliferations were detectable but variable depending on the patient: from a normal range to vastly reduced (Table 2). PHA-induced T cell proliferation was normal in all THES2 patients and in two out of five THES1 patients. With regard to cytokine secretion, pharmacological activation of T cells showed a reduced number of IFN-γ producing T cells in patients compared to healthy individuals (Figure 1D, bottom right panel).

**Table 2:** Evaluation of T cell proliferations after stimulation with mitogens and antigens.

	P1	P2	P3	P4	P5	P6	P7	P8	P9
**Mitogens**									
PHA	Normal	Normal	Normal	Reduced	Reduced	Reduced	nd	Normal	Normal
Anti-CD3	Normal	Normal	Reduced	Very low	Reduced	Very low	nd	Normal	Normal
**Antigens**									
Tetanus toxoid	Positive	nd	Positive	nd	Positive	Negative	nd	Positive	Positive
Tuberculin	Positive	nd	Negative	nd	Positive	Negative	nd	Positive	Negative
Candidin	Positive	nd	Negative	nd	Positive	nd	nd	Negative	nd

*Proliferation after PHA stimulation: normal, above 55%; reduced, between 20 and 55%; very low, between 8 and 20%*.

*Proliferation after CD3 stimulation: normal, above 40%; reduced, between 10 and 40%; very low, between 2 and 10%*.

*nd, not done; PHA, phytohemagglutinin*.

### Hyporesponsiveness of NK Cells in Response to Target Cell or Pharmacological Activation

**Figure 2:**
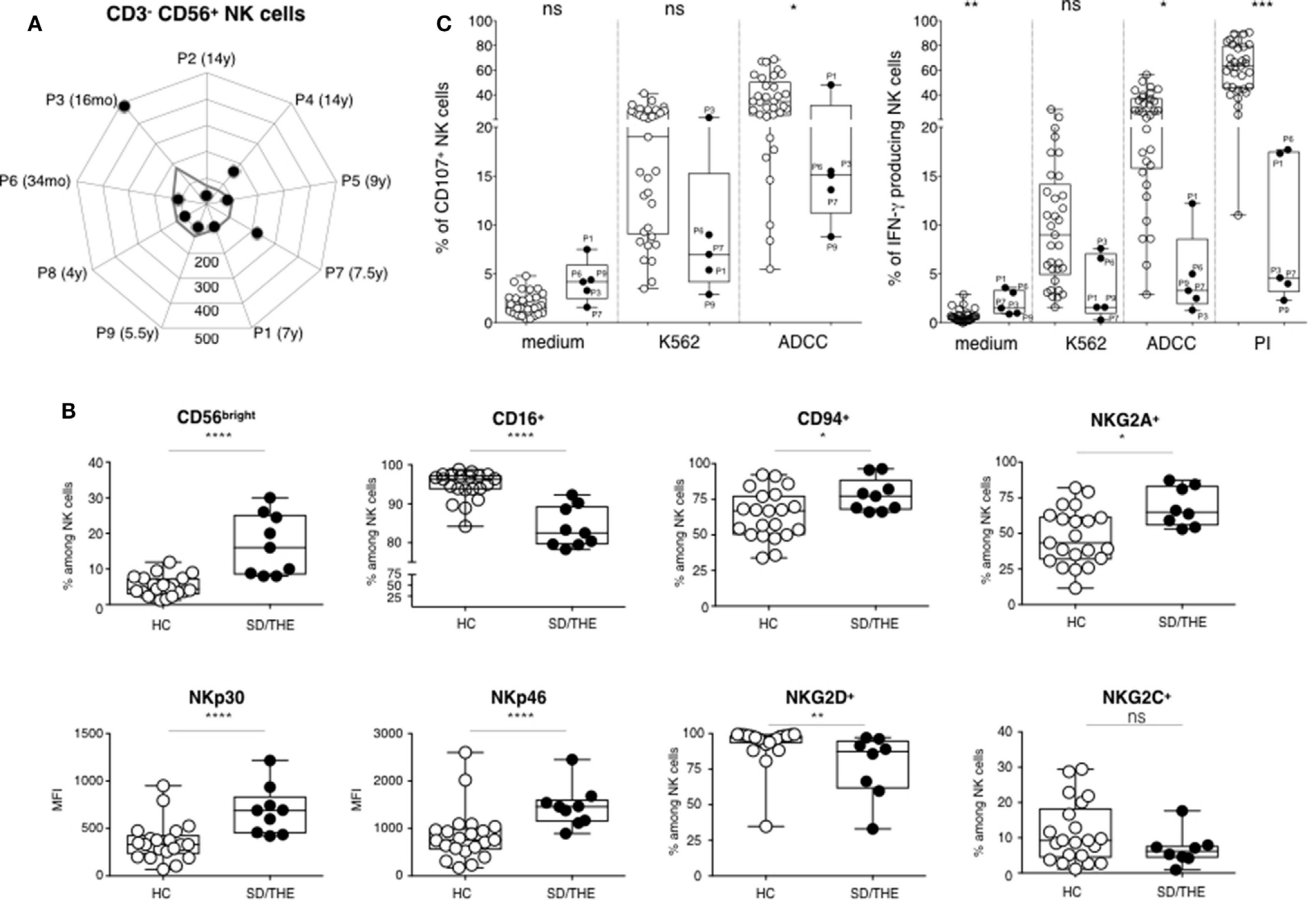
Phenotypic and functional analysis of NK cells. **(A)** Absolute numbers of CD3^−^CD56^+^ NK cells (cells per μl). **(B)** Cell surface expression of the indicated molecules on NK cells among the peripheral blood lymphocytes from healthy controls HC (closed circles) and syndromic diarrhea/trichohepatoenteric syndrome (SD/THE) patients (open circles), given as a percentage of total NK cells or as median fluorescence intensity (MFI). **(C)** NK cell functions evaluated by their ability to release granules and to express CD107 at the cell surface (left panel) and to produce interferon gamma (right panel) after target cell contact or PMA/ionomycin (PI) activation as previously described (9). Healthy controls (*n* = 33) and patients (*n* = 5) are indicated as open circles and closed circles, respectively. Box-plot with medians, 25th–75th percentiles, and min–max values are shown. ns, not significant, *p* > 0.05; *p < 0.05; **p < 0.01; ***p < 0.001; ***p < 0.001; ***p < 0.001 (Mann–Whitney test).

CD3^−^CD56^+^ NK cell counts in SD/THE patients were low but still within normal values (Figure 2A). In the linear differentiation model for human NK cells, CD56^bright^ and CD56^dim^ NK cells corresponded to sequential steps in NK cell differentiation (10). CD56^dim^ NK cells had high surface density of the low-affinity Fc receptor CD16 (CD16^hi^) which accounted for 90% of peripheral blood NK cells. CD56^bright^ NK cells (which are CD16^lo/neg^) accounted for less than 10% of circulating NK cells. CD56^bright^ NK cells proliferated and produced IFN-γ in response to stimulation with cytokines, such as IL-12 and IL-18, whereas CD56^dim^ NK cells were cytolytic and produced cytokines when they encountered target cells.

The CD56^bright^ NK cell subset was over-represented in our patients due to a reduced number of the CD56^dim^ subset (Figure 2B). This led to higher frequency of NKG2A^+^, CD94^+^ NK cells, as well as a higher expression of activating NKp30 and NKp46 receptors in these patients compared to healthy individuals (Figure 2B). NK cells are innate lymphocytes involved in control of viral infection and tumor development. Thus, we further analyzed both the degranulation, which is the first step of the cytotoxic process and the IFN-γ production. We studied the functionality of blood NK cells by PBMCs culture with medium or K562 cells for the evaluation of natural cytotoxicity, and with P815 cells coated with anti-P815 antibodies for the evaluation of the antibody-dependent cellular cytotoxicity (ADCC). NK cell degranulation, represented by the proportion of CD107^+^ NK cells, was reduced in SD/THE patients for the various conditions tested (Figure 2C, left panel). Along the same line, there is reduced number of IFN-γ producing NK cells in patients compared to healthy controls after target cell stimulation or pharmacological activation (Figure 2C, right panel).

## Discussion

Our data confirm that immunological defect is a significant feature of SD/THE patients. Lymphocyte subset analysis showed a normal or slightly reduced number of CD3^+^, CD4^+^, CD8^+^, CD19^+^, and CD3^−^CD56^+^ cells. It was previously reported that SD/THE patients could display normal memory CD19^+^CD27^+^ B cell enumeration (5), which is the case for two out of nine patients in our cohort. By contrast, there was almost an absence of switched memory CD20^+^IgM^−^CD27^+^ B cells in all patients and it could have been responsible for their hypogammaglobulinemia. As a matter of fact, most of them received intravenous or subcutaneous immunoglobulin replacement. That made the evaluation of specific antibody production after vaccination very difficult. Nevertheless, some patients were able to mount antigen-specific immune responses. Regarding pneumococcal immunization, all immunized patients received protein-conjugated antigens while the response to pneumococcal antigens was tested by challenging the patient with the pneumococcal capsular polysaccharides in the 23-valent pneumococcal vaccine (PPV23). This did not allow us to conclude about specific anti-pneumococcal response in our cohort. However, there are hints suggesting a problem with antibody production capacity: more than half the patients had low IgG level, specific antibody production was absent or weak after certain immunizations and B cell switched memory subset was at very low rate.

TTC37 and SKIV2L proteins are involved in RNA degradation. It was recently shown that SKIV2L is a negative regulator of RIG-I-like receptor signaling. The RNA helicase RIG-I binds to RNA of many virus. Interestingly this pathway was reported to be involved in type I IFN induction during EBV and HSV infections. Interestingly, patients with deficiencies in Mcm4 and Gins1, which are involved in DNA helicase complexes (11, 12), have a profound NK cell lymphopenia. In addition, NK cell deficiencies are frequently associated with higher susceptibility to herpes virus infections and four out nine SD/THE patients had history of EBV infection. Thus, we carried out the first phenotypic and functional evaluation of NK cells in these patients. NK cell lymphopenia was found in six patients out of nine patients due to a small number of the mature CD56^dim^ NK cell subset. Besides these abnormal counts, NK cells were also hyporesponsive to target cell or pharmacological activations: both degranulation and number of IFN-γ producing NK cells were reduced in most patients. Interestingly, the number of IFN-γ producing T cells was also reduced when stimulated by PMA and ionomycin.

Because of this defect and the role of TTC37 and SKIV2L proteins in RNA surveillance and degradation, these patients could be prone to infections by RNA virus. So far, only one measles death has been reported in a 18-year-old patient (7). However, over half of the nine patients have developed severe EBV infections. It remains an interesting possibility that hyporesponsiveness of NK cells combined to this T cell defect can be implicated in the occurrence of hemophagocytosis which was noted in four patients in this cohort. These transient hemophagocytosis were related to EBV infections and were not inherited forms of hemophagocytic lymphohistiocytosis in which there is an accumulation of CD8^+^ T cells associated with increased secretion of IFN-γ. It is worth noting that hemophagocytosis was restricted to STHE1 patients (four out of six). Although there is no association between the intensity of the NK cell defects and the type of mutation, abnormal T cell proliferations were found only in STHE1 patients. As previously suggested, NK cell could be redundant in natural conditions if T cells and B cells are present and functional (13). Our work suggests that it is of interest to monitor T and B cell subsets as well as NK cell functions in SD/THE patients since an alteration of the adaptive immunity could emphasize the impact of this NK cell defect in the control of some viral infections, such as EBV infections.

Low class-switched B cell count has been associated with a lot of diagnosis and particularly in *MAP3K14* deficiency in which a low NK cell number and function was also reported (14). Altogether, this work highlights the need for an immunological evaluation in SD/THE patients. In addition, this is the first report of phenotypic and functional NK cell defects in these patients. Finally, further studies are necessary to understand what are the molecular mechanisms by which *TTC37* and *SKIV2L* mutations lead to such immunological disorders.

## Methods

### Cells and Antibodies

Total lymphocyte, CD4^+^T cell, CD8^+^ T cell, and CD19^+^ B cell populations were quantified with 6-Color BD Multitest and BD Trucount Technologies according manufacturer’s instructions. NK cells were defined as CD3^−^CD56^+^ cells within the lymphocyte size/structure gate. The following monoclonal antibodies were used: anti-CD27 (IgG1, M-T271), anti-IgD (IgG2a, IA6-2), anti-IgM (IgG1, G20–127), anti-CD56 (IgG1, B159), anti-CD3 (IgG1, SK7), anti-CD16 (IgG1, 3G8), anti-CD94 (IgG1, HP-3D9), from Becton Dickinson, San Diego, CA, USA; anti-NKp30 (IgG1, AZ20), anti-NKp46 (IgG1, 9E2), anti-NKG2A (IgG2b, Z199), and anti-NKG2D (IgG1, 1D11), from Beckman Coulter, Villepinte, France; and anti-NKG2C (IgG1, Fab 138C) from R&D Systems, Abingdon, UK. Data acquisition and analysis were performed by using a BD FACSCanto II cytometer and FlowJo software (Becton Dickinson, Le Pont de Claix, France), respectively.

Fresh human PBMCs were isolated by Ficoll-Hypaque density gradient centrifugation (GE Healthcare), from heparin-treated whole-blood samples obtained from patients or healthy volunteers.

### NK Cell Degranulation and IFN-γ Production by T and NK Cells

NK cell activation was monitored by assessing the ability of these cells to degranulate and to produce interferon gamma in response to several stimuli: MHC class I-negative human erythroleukemic K562 target cells, to evaluate natural cytotoxicity; mouse mastocytoma P815 cells coated with rabbit anti-mouse lymphocyte antibodies (Accurate Biochemicals, Westbury, NY, USA), to mimic ADCC and pharmacological activation with PMA and ionomycin as previously described (9). The enumeration of T and NK cells able to produce IFN-γ was analyzed by intracellular staining after PMA and ionomycin stimulation. Data acquisition and analysis were performed by using a BD FACSCanto II cytometer.

### Analysis of T Cell Proliferations

CFSE-stained PBMCs were cultured for either 3 days (mitogen activation) or 7 days (antigen activation). Proliferation was defined as the level of CFSE-dilution within T cell gate after stimulation. Mitogen concentrations were 10 µg/ml PHA (Oxoid), and 10 ng/ml anti-CD3 (OKT3, eBiosciences). Antigen concentrations were 5 µg/ml tuberculin (Statens, Denmark), 5 µg/ml candidin (Biorad), and 0.9 Lf/ml tetanus toxoid (Statens, Denmark). CFSE-dilution was quantified by using a BD FACSCanto II cytometer.

## Ethics Statement

A written informed consent was obtained from each participant for the publication of this case report. This study was performed according French rules (Art. L. 1243-1 et Art. L. 1245-2 du Code de la Santé Publique).

## Author Contributions

FV, VB, CF, and AF: analysis and interpretation of data for the work; drafting the work. EM, M-EC, BD, ED, JL, CM-V, NP, AP, OG, J-PH, PB, and CB: substantial contributions to the conception or design of the work.

## Conflict of Interest Statement

The authors declare that the research was conducted in the absence of any commercial or financial relationships that could be construed as a potential conflict of interest.
